# High-frequency rTMS over bilateral primary motor cortex improves freezing of gait and emotion regulation in patients with Parkinson’s disease: a randomized controlled trial

**DOI:** 10.3389/fnagi.2024.1354455

**Published:** 2024-01-24

**Authors:** Wenjing Song, Zixuan Zhang, Bingchen Lv, Jinyu Li, Hao Chen, Shenyang Zhang, Jie Zu, Liguo Dong, Chuanying Xu, Manli Zhou, Tao Zhang, Ran Xu, Jienan Zhu, Tong Shen, Su Zhou, Chenchen Cui, Shuming Huang, Xi Wang, Yujing Nie, Kainat Aftab, Qihua Xiao, Xueling Zhang, Guiyun Cui, Wei Zhang

**Affiliations:** ^1^Department of Neurology, The Affiliated Hospital of Xuzhou Medical University, Xuzhou, Jiangsu, China; ^2^Department of Neurology, The First Clinical College, Xuzhou Medical University, Xuzhou, Jiangsu, China; ^3^Department of Neurology, Fujian Medical University Union Hospital, Fuzhou, Fujian, China; ^4^Department of Neurology, The Affiliated Suqian Hospital of Xuzhou Medical University, Suqian, Jiangsu, China; ^5^Department of Neurology, Suining County People’s Hospital, Xuzhou, Jiangsu, China

**Keywords:** transcranial magnetic stimulation, primary motor cortex, freezing of gait, emotion regulation, Parkinson’s disease

## Abstract

**Background:**

Freezing of gait (FOG) is a common and disabling phenomenon in patients with Parkinson’s disease (PD), but effective treatment approach remains inconclusive. Dysfunctional emotional factors play a key role in FOG. Since primary motor cortex (M1) connects with prefrontal areas via the frontal longitudinal system, where are responsible for emotional regulation, we hypothesized M1 may be a potential neuromodulation target for FOG therapy. The purpose of this study is to explore whether high-frequency rTMS over bilateral M1 could relieve FOG and emotional dysregulation in patients with PD.

**Methods:**

This study is a single-center, randomized double-blind clinical trial. Forty-eight patients with PD and FOG from the Affiliated Hospital of Xuzhou Medical University were randomly assigned to receive 10 sessions of either active (*N* = 24) or sham (*N* = 24) 10 Hz rTMS over the bilateral M1. Patients were evaluated at baseline (T0), after the last session of treatment (T1) and 30 days after the last session (T2). The primary outcomes were Freezing of Gait Questionnaire (FOGQ) scores, with Timed Up and Go Test (TUG) time, Standing-Start 180° Turn (SS-180) time, SS-180 steps, United Parkinson Disease Rating Scales (UPDRS) III, Hamilton Depression scale (HAMD)-24 and Hamilton Anxiety scale (HAMA)-14 as secondary outcomes.

**Results:**

Two patients in each group dropped out at T2 and no serious adverse events were reported by any subject. Two-way repeated ANOVAs revealed significant group × time interactions in FOGQ, TUG, SS-180 turn time, SS-180 turning steps, UPDRS III, HAMD-24 and HAMA-14. Post-hoc analyses showed that compared to T0, the active group exhibited remarkable improvements in FOGQ, TUG, SS-180 turn time, SS-180 turning steps, UPDRS III, HAMD-24 and HAMA-14 at T1 and T2. No significant improvement was found in the sham group. The Spearman correlation analysis revealed a significantly positive association between the changes in HAMD-24 and HAMA-14 scores and FOGQ scores at T1.

**Conclusion:**

High-frequency rTMS over bilateral M1 can improve FOG and reduce depression and anxiety in patients with PD.

## Introduction

Parkinson’s disease (PD) is one of the most common degenerative diseases of the nervous system in the middle-aged and elderly. Clinically, it is characterized by delayed movement, static tremor, muscle rigidity, and postural gait disorders (Postuma et al., 2015; Erro and Stamelou, 2017; Jankovic and Tan, 2020). Freezing of Gait (FOG) is a prevalent and severely debilitating gait disorder observed in the middle and advanced stages of PD patients. It mainly presents with hesitation to start walking, and patients often experience a sensation of their feet being firmly rooted to the ground, typically lasting for several seconds. In severe instances, walking inability may occur (Gao et al., 2020). The occurrence of FOG is typically observed during initiation, turning, navigating narrow spaces, and approaching a target. Environmental factors, emotional cognition, and other variables exert significant influence on FOG (Spildooren et al., 2019; Ehgoetz Martens et al., 2020; Weiss et al., 2020). The prevalence of FOG increases with disease progression, as evidenced by a 12-year longitudinal study reporting a substantial incidence rate of 63% in PD patients with an illness duration exceeding 10 years (Forsaa et al., 2015). Furthermore, FOG poses elevated risks for falls and fractures while also giving rise to adverse psychological issues such as anxiety and depression, thereby severely impacting the patient’s quality of life (Bekkers et al., 2017; Okuma et al., 2018; Witt et al., 2019).

Due to the heterogeneous clinical manifestations and underlying pathophysiological mechanisms associated with FOG, pharmacological interventions and deep brain stimulation (DBS) are not considered optimal for its amelioration. In recent years, increasing attention has been directed toward the unique efficacy of physical therapy in addressing FOG (Dagan et al., 2018; Gilat et al., 2018; Cosentino et al., 2020; Delgado-Alvarado et al., 2020). Repetitive transcranial magnetic stimulation (rTMS), a non-invasive neuroregulation technique based on the principle of magnetic induction, induces current flow and action potentials within the cerebral cortex through pulsed magnetic fields. This can modulate brain metabolism and related electrophysiological activities (Burke et al., 2019; Lefaucheur, 2019).

In 2020, the latest European clinical guidelines recommended rTMS over the bilateral primary motor cortex (M1) as a Grade-B recommendation for improving motor function in PD (Burke et al., 2019; Lefaucheur et al., 2020). The efficiency of this approach has been confirmed by multi-center clinical trials and meta-analyses (Burke et al., 2019; Lefaucheur et al., 2020). Li et al.’s study (Li et al., 2018), based on resting-state functional magnetic resonance imaging (fMRI), found synchronous decreases in spontaneous neural activity within the bilateral M1 region and subparietal lobule of the brain in patients with PD-related FOG (PD-FOG). Furthermore, according to the “decoupling model” (Plotnik et al., 2012), it is suggested that patients with FOG have difficulty initiating or experiencing freezing due to impaired integration between M1-mediated walking initiation and postural adjustment mediated by the supplementary motor area (SMA) (Nomura and Kirimoto, 2018; de Lima-Pardini et al., 2020; Tsuru et al., 2020). Previous studies have reported that high-frequency rTMS applied to M1 can enhance the local excitability of the primary motor cortex and indirectly activate SMA neural activity (Bestmann et al., 2003). As suggested previously (Kim et al., 2015), high frequency rTMS over the lower leg primary motor cortex of the dominant hemisphere may be an effective treatment for alleviating FOG in PD. Furthermore, FOG usually manifests as problems with bilateral coordination between the legs. Therefore, we hypothesize that targeting the bilateral M1 region with rTMS will get a better therapy response for improving FOG in PD.

Additionally, anxiety and depression have been associated with FOG and the M1, premotor cortex, and prefrontal regions are interconnected through the frontal longitudinal system (FLS) (Komaitis et al., 2019). While some clinical studies have reported the effects of motor cortex rTMS on motor symptoms in PD, limited research exists exploring its effects on non-motor symptoms, such as anxiety, depression, and cognition function. Thus, our main objective was to investigate the clinical efficiency of high-frequency rTMS targeting the bilateral M1 for treating FOG in PD while also observing its effects on walking ability, overall motor function, anxiety, depression, and cognition among these patients. In addition, previous studies have limitations of small sample sizes. The present study recruited more subjects in the rTMS group than other research. This study will provide new evidence of high-frequency rTMS over the bilateral M1 serving as an therapy for alleviating FOG in PD patients.

## Materials and methods

### Participants

Forty-eight PD-FOG patients, who attended the PD Center of the Affiliated Hospital of Xuzhou Medical University from December 2019 to March 2023, were recruited. The inclusion criteria were (a) age between 45 and 80 years old; (b) compliance with the 2015 MDS version of the PD diagnostic criteria (Postuma et al., 2015); (c) the determination criteria for FOG (≥ any one of the following) (Mancini et al., 2019).

(i) Subjective decision: according to item 3 of the Freezing of Gait Questionnaire (FOGQ) (Giladi et al., 2000; Gal et al., 2020), patients were asked whether they felt their feet were stuck to the ground during starting, turning, or walking; (ii) subjective determination: confirmed by showing typical videos of a typical freezing gait episode to the patient and caregivers (Nonnekes et al., 2017); (iii) Objective determination: freezing gait was induced by bi-directional 360° rapid turns, dual-tasking, and small rapid steps (van Dijsseldonk et al., 2018; Mancini et al., 2019); (d) Hoehn-Yahr stage (H-Y stage) (Hoehn and Yahr, 1967; Skorvanek et al., 2017): stage 2–4; (e) Mini-Mental State Examination (MMSE) (Santana et al., 2016) ≥ 24 points; (f) PD medication stabilized for more than 2 weeks and no change in medication regimen during the study period; and (g) being available to cooperate in completing the rTMS treatment and symptom assessment. The study protocol was approved and supervised by the Affiliated Hospital of Xuzhou Medical University Ethics Committee (No. XYFY2019-KL205-02). All patients had agreed and confirmed their informed consent prior to the study. The present study was registered at the Chinese Clinical Trial Registry (Registration number: ChiCTR2000031717).^1^

### Experimental design

This study was a single-center, randomized, double-blind and sham-controlled trial in which 48 subjects were randomly assigned (with 1:1 ratio) via sealed envelopes into two groups, one group receiving 10 Hz rTMS (*N* = 24) and the other group receiving sham stimulation (*N* = 24). Both subjects and researchers were blind to the randomization group, and only the clinician responsible for the rTMS treatment was unmasked to the randomization sequence.

### rTMS and sham protocols

The subjects in this study were treated with rTMS during the drug “on” period at approximately the same time each day, using a transcranial magnetic stimulator, MagstimRapid2, which had a figure-of-eight coil with a dynamic air-cooling device. The resting motor threshold (RMT) was measured in all subjects prior to rTMS. Here, RMT is defined as the ability to evoke a visible voluntary contraction of the target muscle, the thenar muscles of the right hand, in 50% of successive trials. In the rTMS group, the stimulation sites were the bilateral M1.

The coil is parallel to the tangent plane of the target during stimulation (the direction of the magnetic field lines is perpendicular to the tangent plane of the target). The brain area on the side with more severe motor symptoms was stimulated first, followed by the contralateral side. The stimulation frequency was 10 Hz, the stimulation intensity was 90% of the resting motor threshold (RMT). The RMT is defined as the minimum intensity to evoke a visible voluntary contraction of the target muscle. The RMT in the rTMS and sham group were 57.3% ± 17.5 and 56.8% ± 15.2% respectively, with a stimulus intensity of 51.5% ± 15.8 and 51.1% ± 13.7%, respectively. The string stimulation time was 5 s, the inter-string interval was 55 s, and the total number of pulses was 1,000 pulses/trial, which was performed once a day for 40 min, and the stimulation lasted for 10 days. During the stimulation of the sham group, the figure-of-eight coils were placed perpendicular to the tangent plane of the target point (the direction of the magnetic field lines was parallel to the tangent plane of the target point), and the target point of the stimulation and its parameters were the same as those in the treatment group. Previous studies have reported that a 90° rotation of the stimulation coil is able to produce similar sounds and sensations as active stimulation, which significantly reduces the strength of the magnetic field in the target area and brings it closer to zero (Kim et al., 2015). All subjects were scheduled to complete the trial at different times to avoid discussing with each other as this could have affected the realization of blinding during data collection.

### Clinical assessment

**Figure 1:**
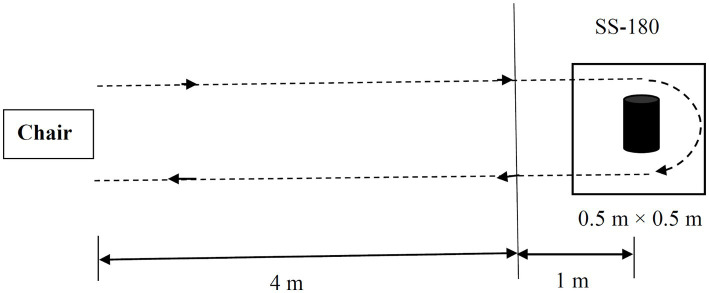
Schematic drawing of the standard Timed Up and Go (TUG) task and the modified Standing Start 180° Turn Test (SS-180). The participants were instructed to walk for a distance of 5 m from their seat and come back to a chair after turning 180° around a cylindrical obstacle in a 0.5 m × 0.5 m target box. The turn time and turning steps during the 180° turn are measured from the point when the participant is 1 m from the obstacle. The whole walking tasks were recorded by a video camera.

Subjects underwent clinical assessments at baseline (T0), after the tenth treatment session (T1) and 30 days after T1. Assessments were conducted in the practically “ON” state, to the extent possible, during the same time period each day. All participants were instructed to perform a standard Timed Up and Go (TUG) task and a modified Standing-Start 180° Turn Test (SS-180) (Figure 1; Kim et al., 2015). The TUG and SS-180 task were repeated twice in each direction. The whole process is recorded by a video camera and the video analysis were independently performed by a blinded rater. The mean time and number of steps were calculated as the final result. The primary outcome was the FOGQ score, and the secondary outcomes were the total TUG time, SS-180 turn time, SS-180 turning steps, with the United Parkinson Disease Rating Scales (UPDRS) III score used to evaluate the effect of rTMS on global motor function. Furthermore, MMSE, MoCA and FAB scores were used to observe the effect of rTMS on cognitive function, with HAMD-24 and HAMA-14 scores utilized to observe the effect of rTMS on depression and anxiety symptoms.

### Statistical analysis

In this study, data were analyzed using SPSS 23.0 software (IBM, Chicago, IL, USA). Demographic data was presented as mean ± SD for continuous variables and as ratios or percentages for categorical variables. Independent two samples *t*-test was used to compare continuous variables, and the *χ^2^*-test was performed for the comparison of categorical variables. Two-way repeated ANOVA, with Group (rTMS / sham group) as between-subject factor and Time (T0, T1, T2) as within-subject factor, was employed to analyze the effects of rTMS on the clinical outcomes. The threshold for the level of significance was set at *α* = 0.05. Spearman correlation analysis was performed to investigate the association between the changes in HAMD-24 and HAMA-14 scores and FOGQ scores. A bilateral *p*-value <0.05 was considered statistically significant.

## Results

### Participants

The demographic and clinical characteristics of the subjects are presented in Table 1. Two patients from each group dropped out during the T2 follow-up, resulting in a total of 44 patients included in the analysis. There were no significant differences between the TMS and sham stimulation groups at baseline for gender, age, course of disease, H&Y stage, FOGQ, UPDRS III, LEDD, MMSE, MoCA, FAB, HAMD-24 and HAMA-14.

**Table 1:** Demographic and clinical characteristics of participants.

Variables		rTMS group (*n* = 22)	Sham group (*n* = 22)	*χ^2^/t*	*P*
Gender (M/F)		15/7	13/7	0.393	0.531
Age		67.36 ± 6.99	70.50 ± 6.76	−1.513	0.138
Disease duration		6.18 ± 1.62	6.77 ± 2.02	−1.069	0.291
H&Y				0.10^a^	0.75
	2–2.5	15 (68.20%)	14 (63.60%)	/	/
	3	7 (31.80%)	8 (36.40%)	/	/
FOGQ		13.41 ± 5.11	13.55 ± 4.42	−0.095	0.925
UPDRS III		41.68 ± 12.96	43.23 ± 10.73	−0.431	0.669
LEDD (mg/d)		706.82 ± 236.03	741.23 ± 197.97	−0.524	0.603
MMSE		26.45 ± 1.71	25.91 ± 1.41	1.154	0.255
MoCA		21.09 ± 4.23	20.36 ± 3.98	0.587	0.560
FAB		11.45 ± 2.60	11.59 ± 2.61	−0.174	0.863
HAMD-24		14.82 ± 6.61	15.18 ± 6.21	−0.188	0.852
HAMA-14		9.32 ± 4.36	10.82 ± 4.91	−1.071	0.290

rTMS, repetitive transcranial magnetic stimulation; M, male; F, female; H&Y, Hoehn & Yahr; FOGQ, freezing of gait questionnaire; UPDRS III, unified Parkinson’s disease rating scale part III; LEDD, levodopa-equivalent daily dose; MMSE, mini-mental state examination; MoCA, Montreal cognitive assessment; FAB, frontal assessment battery; HAMD-24, Hamilton depression rating scale-24 item; HAMA-14, Hamilton anxiety rating scale-14 item.

### Clinical efficacy: primary outcome

**Figure 2:**
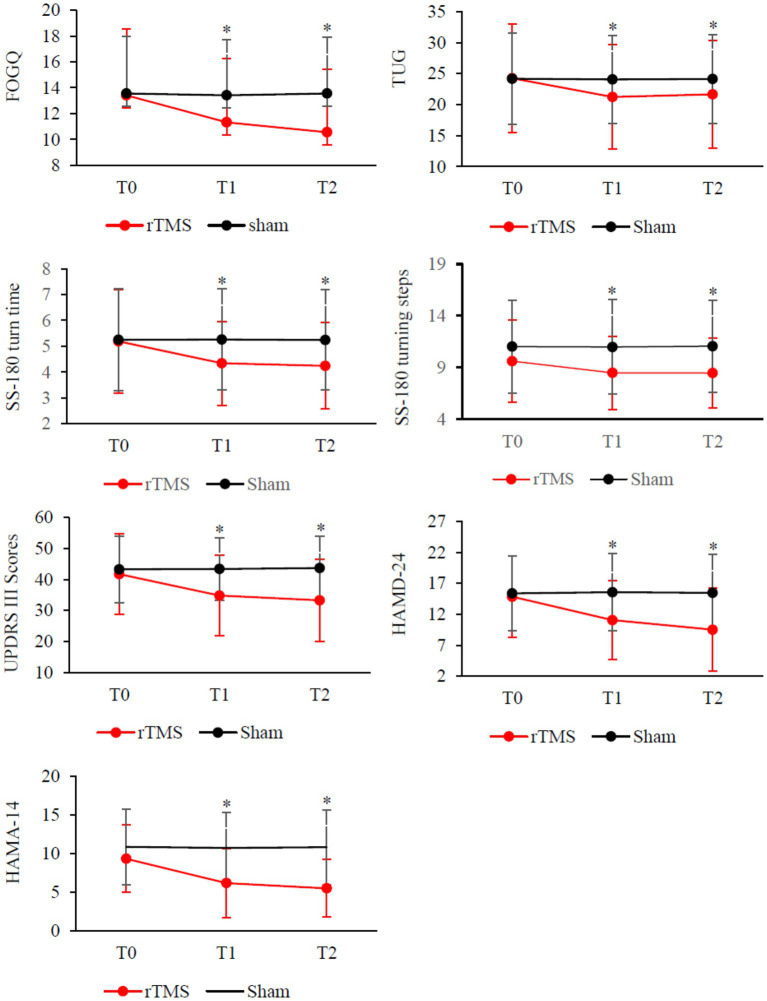
Clinical changes after rTMS therapy, including FOGQ, TUG, SS-180 turn time, SS-180 turning steps, UPDRS-III scores, HAMD-24 and HAMD-14. Red, rTMS group; black, sham group. *Post-hoc analysis shows a significant difference as compared to the baseline (T0) in the group.

As shown in Table 2 and Figure 2, there was a significant Group × Time interaction (*p* < 0.001) as well as significant Time (p < 0.001) main effects. Post-hoc analysis revealed that FOGQ scores significantly improved at T1 and T2 compared to T0 for the rTMS group but not for the sham group.

**Table 2:** Clinical efficiency of the rTMS and Sham group.

	rTMS group	Sham group		DF	F	*P*
FOGQ						
T0	13.41 ± 5.11	13.55 ± 4.42	Group	1.000	1.557	0.219
T1	11.32 ± 4.91	13.41 ± 4.31	Time	1.392	49.959	0.000
T2	10.55 ± 4.85	13.55 ± 4.35	Group × Time	1.392	47.311	0.000
TUG						
T0	24.24 ± 8.76	24.17 ± 7.33	Group	1.000	0.539	0.467
T1	21.23 ± 8.42	24.05 ± 7.12	Time	1.741	19.894	0.000
T2	21.65 ± 8.72	24.12 ± 7.11	Group × Time	1.741	17.473	0.000
SS-180 turn time						
T0	5.19 ± 2.00	5.25 ± 1.98	Group	1.000	1.422	0.240
T1	4.34 ± 1.62	5.26 ± 1.96	Time	1.228	16.877	0.000
T2	4.24 ± 1.67	5.24 ± 1.94	Group × Time	1.228	16.342	0.000
SS-180 turning steps						
T0	9.60 ± 4.01	11.01 ± 4.47	Group	1.000	3.146	0.083
T1	8.47 ± 3.55	10.97 ± 4.58	Time	1.412	7.486	0.004
T2	8.45 ± 3.40	11.05 ± 4.46	Group × Time	1.412	7.354	0.004
UPDRS III						
T0	41.68 ± 12.96	43.23 ± 10.73	Group	1.000	3.764	0.059
T1	34.77 ± 13.08	43.36 ± 10.11	Time	1.574	72.308	0.000
T2	33.23 ± 13.17	43.64 ± 10.21	Group × Time	1.574	84.738	0.000
HAMD-24						
T0	14.82 ± 6.61	15.36 ± 6.08	Group	1.000	3.814	0.058
T1	11.05 ± 6.42	15.55 ± 6.21	Time	1.642	29.802	0.000
T2	9.50 ± 6.67	15.45 ± 6.19	Group × Time	1.642	32.661	0.000
HAMA-14						
T0	9.32 ± 4.36	10.86 ± 4.91	Group	1.000	8.213	0.006
0.006 T1	6.18 ± 4.22	10.73 ± 4.55	Time	1.578	55.370	0.000
T2	5.50 ± 3.70	10.82 ± 4.82	Group × Time	1.578	55.127	0.000

rTMS, repetitive transcranial magnetic stimulation; FOGQ, freezing of gait questionnaire; TUG, Timed-up and go test; SS180, Standing-start 180° turn test; UPDRS III, unified Parkinson’s disease rating scale part III; HAMD-24, Hamilton depression rating scale-24 item; HAMA-14, Hamilton anxiety rating scale-14 item.

### Clinical efficacy: secondary outcomes

Our results demonstrate that compared to the sham stimulation, TMS led to significant Group × Time interactions on measures such as TUG time (*p* < 0.001), SS-180 turn time (*p* > 0.001), SS180 turn steps (*p* < 0.01), UPDRS III score (*p* < 0.01), HAMD-24 score (*p* > 0.01), and HAMA-14 score (*p* = 0.001). The analysis revealed significant improvements in T1 and T2 compared to the baseline (T0) on the turn times of SS-180, the number of steps taken during SS-180 turns, UPDRS III scores, HAMD-24 scores, and HAMA-14 scores. No significant Group × Time interactions were found when comparing MMSE or MoCA scores.

### The Spearman correlation analysis revealed a significant positive association between the changes in HAMD-24 and HAMA-14 scores (△HAMD-24 and △HAMA-14) and FOGQ scores (△FOGQ) before and after intervention in the treatment group

**Figure 3:**
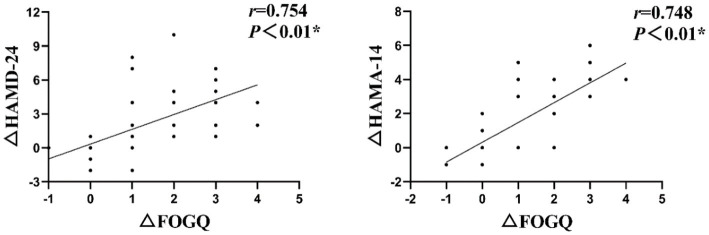
The Spearman correlation analysis revealed a significant positive association between the changes in HAMD-24 and HAMA-14 scores (△HAMD-24 and △HAMA-14) and FOGQ scores (△FOGQ) at T1.

Specifically, a positive correlation was observed between the improvements in depression and anxiety scores before and after intervention, with corresponding improvements in FOGQ scores at T1 (Figure 3) (*r* = 0.754, *p* < 0.01; *r* = 0.748, *p* < 0.01).

### Adverse events

Minor and transient adverse events were rarely reported during stimulation sessions. In one instance, a patient receiving rTMS reported mild dizziness while another reported a mild headache after their first session. In the sham group, only one patient reported scalp numbness.

## Discussion

This randomized, double-blind, and sham-controlled study demonstrated that high-frequency rTMS stimulation over the bilateral M1 can lead to a short-lasting improvement in FOG in patients with PD. Additionally, there was some relief observed in the patients’ motor symptoms as well as anxiety and depression symptoms. Notably, the FOG improvement was found to be associated with a reduction in anxiety and depression and the effects of high-frequency rTMS on FOG were cumulative and continued for at least 30 days after treatment. Our findings suggest that high-frequency rTMS over the bilateral M1 may be considered a feasible treatment option for FOG in PD.

In recent years, rTMS has had widespread usage as a non-invasive neuromodulation technique for treating various conditions, including PD, stroke, depression, and other neuropsychiatric disorders (Guo et al., 2017; Dionísio et al., 2018; Latorre et al., 2019; Leung et al., 2020). While several studies both domestically and internationally, have reported the effects of motor cortex rTMS on motor symptoms of PD (Chou et al., 2015; Yang et al., 2018; Latorre et al., 2019), only two clinical reports exist regarding the use of FOGQ as an observation index for assessing the impact of M1 rTMS on FOG in PD (Rektorova et al., 2007; Kim et al., 2015).

Rektorova et al. (2007) were among the first to report on the efficacy of rTMS for alleviating FOG symptoms in PD patients using a cross-over experimental design. However their findings indicated that stimulating the left M1 area or left DLPFC did not improve FOGQ scores, which contradicts our positive results. According to previous studies based on the “threshold model” theory (Plotnik et al., 2012) along with numerous others (Kleiner et al., 2018; Son et al., 2018; Spildooren et al., 2019), it is understood that FOG is linked to asymmetry and coordination issues within gait patterns involving both lower limbs; clinically it often presents itself through more severe unilateral symptoms. In Rektorova et al.’s study, only the left motor cortex was stimulated, and the researchers did not report the side with more severe lower limb motor symptoms in 4 subjects.

The study by Rektorova et al. included only four subjects, which may have introduced bias into the results. Furthermore, Kim et al. (2015) suggested that rTMS treatment can enhance cortical excitability in PD-FOG and this effect can last for 1 week after treatment, indicating that rTMS may lead to long-term improvement of FOG through cumulative increase in synaptic efficacy. These findings are consistent with our study, demonstrating that high-frequency rTMS in the M1 region can improve FOG symptoms in PD patients.

Importantly, our research results showed a significant decrease in the FOGQ score at T2 compared to T1, suggesting there can be a long-term positive effect (at least 30 days) of high-frequency rTMS on FOG (Latorre et al., 2019). Meta-analysis has shown that the effectiveness and duration of rTMS depend on factors such as total number of pulses, number of stimuli, and interval (Chou et al., 2015), with Bäumer et al. (2003) having demonstrated cumulative changes in cortical excitability with multiple consecutive sessions of rTMS. Therefore, multi-temporal high-frequency rTMS could achieve sustained effects by repeatedly inducing synaptic plasticity and promoting synaptic long-term potentiation from transient to more lasting states.

The mechanism underlying rTMS treatment for FOG in PD remains unclear, but it may be associated with alterations in related neural pathways and neurotransmitters. High-frequency rTMS applied to the M1 region might directly influence striatal activity by activating the motor cortex-thalamus-basal ganglia pathway and subsequently modulating inhibitory impulses within the medial globus pallidus to improve FOG (Kim et al., 2015). Similarly, positron emission computed tomography (PET) studies have indicated (Strafella et al., 2005) that high frequency (10 Hz) rTMS applied to M1 could induce local release of endogenous dopamine within the ipsilateral putamen via cortico-basal ganglia circuits, this is consistent with anatomical projections observed previously. Khedr et al. (2007) treated 20 patients with moderate to severe PD with high frequency (25 Hz) rTMS in the M1 region once a day for 6 consecutive days, with 3,000 pulses per time. Their study found that the serum dopamine level of PD patients was significantly higher than the baseline after all treatments, which was related to the improvement of clinical motor symptoms.

Mi et al. (2020) found that high-frequency rTMS over the bilateral SMA could effectively improve gait disorders by improving the abnormal brain network connectivity pattern related to FOG symptoms in PD patients and making them more similar to people without PD. It has been reported that high-frequency rTMS in the M1 region can increase the blood oxygen level-dependent signal of SMA, that is, indirectly activate the neural activity of SMA (Bestmann et al., 2003). Therefore, we hypothesize that high frequency rTMS in the M1 region in this study may have promoted synaptic activation of SMA by enhancing the interaction between functionally connected SMA-M1, and then improving the freezing gait by normalizing the brain functional connectivity pattern.

In our research, significant reduction of the UPDRS III score was observed following high-frequency rTMS stimulation over the bilateral M1, indicating that this method may ameliorate patients’ motor symptoms. A meta-analysis conducted by Zanjani et al. (2015) evaluated the efficacy of M1 rTMS on motor symptoms in PD patients and revealed that short-term M1 rTMS treatment significantly diminished UPDRS III scores compared to sham stimulation, which is in line with our findings. Both high-frequency and low-frequency rTMS applied to the M1 region potentially induce transient release of endogenous dopamine in the striatum, thereby exerting an immediate impact on PD motor symptoms (Latorre et al., 2019). Subsequently, Kim et al. (2018) investigated the efficacy of various rTMS frequencies on motor function in PD and observed significant UPDRS III score reduction following both high-frequency and low-frequency stimulation treatments. However, the improvement was more pronounced in the high-frequency treatment group, and the effect was maintained for only 1-month post-rTMS in this group. Both high and low frequency rTMS on M1 have the potential to enhance PD patients’ motor function, albeit with high frequency rTMS being more efficacious in mitigating motor symptoms and demonstrating a longer-term positive impact.

In the present study, no statistically significant changes were observed in MMSE and MoCA scores across different time points in either the treatment or control group, suggesting that high-frequency rTMS over the bilateral M1 fails to enhance cognitive function in PD-FOG. Interestingly, the scores of HAMD-24 and HAMA-14 were significantly improved at both T1 and T2 time points in the rTMS group compared to T0 time points. To date, no reports have been published on the efficacy of M1 rTMS in relation to PD-FOG mood disorders.

Anxiety independently contributes to severity of FOG in people with PD (Pimenta et al., 2019) and depressive symptoms may increase the risk of future development of FOG (Herman et al., 2019). In this study, high-frequency stimulation of the M1 region led to an improvement in depression and anxiety symptoms, and the reduction of depression and anxiety scores was significantly correlated with the improvement of the FOGQ. We postulate that the alleviation of depression and anxiety may contribute to the improvement of PD-FOG symptoms, which is consistent with previous research (Cui and Lewis, 2021). Mood disorders are primarily associated with the function of the prefrontal cortex (Samara et al., 2018; Xu et al., 2019), and the M1, premotor cortex, and prefrontal regions are interconnected through the FLS (Komaitis et al., 2019), which might play a role in integrating the activity of local networks within different frontal lobes. The total sessions of rTMS in this study were 10. It is uncertain whether more sessions will result in better treatment effects since few existing study designs have more than 10 sessions (Deng et al., 2022). Further research is required to establish optimal study design that may induce potentially more beneficial outcomes and this may require trying different combinations of stimulation targets.

This study corroborated the significant amelioration of gait freezing, walking ability, global motor function, and depression and anxiety symptoms in PD patients by administering high-frequency rTMS over the bilateral M1. Our results provide powerful evidence of high-frequency rTMS serving as a safe and effective add-on therapy for improving FOG and reducing anxiety and depression symptoms, and also enrich the therapeutic mechanism from a new perspective. However, several limitations should be considered. Firstly, symptom assessment and rTMS treatment were conducted during the “on” phase of the study, due to the inconvenience of outpatients attending the hospital during a fixed “off” phase daily. Consequently, this study’s findings convey the adjunctive efficacy of rTMS in addition to conventional drug therapy. Secondly, the integration of a neuronavigation positioning system, if feasible, could enhance the accuracy of target stimulation and site adjustments in the current rTMS target localization. Thirdly, the sample size of this study was modest and only monitored the subjects for 30 days post-treatment. Nonetheless, the efficacy of rTMS might persist for up to 3 months or even longer. Future research should incrementally enlarge the sample size and explore the long-term implications of rTMS on FOG.

## Data availability statement

The raw data supporting the conclusions of this article will be made available by the authors, without undue reservation.

## Ethics statement

The studies involving humans were approved by the Affiliated Hospital of Xuzhou Medical University Ethics Committee. The studies were conducted in accordance with the local legislation and institutional requirements. The participants provided their written informed consent to participate in this study.

## Author contributions

WS: Conceptualization, Writing – original draft. ZZ: Data curation, Methodology, Writing – original draft. BL: Methodology, Writing – original draft. JL: Methodology, Writing – original draft. HC: Supervision, Writing – original draft. ShZ: Supervision, Writing – original draft. JZu: Supervision, Writing – original draft. LD: Supervision, Writing – original draft. CX: Supervision, Writing – original draft. MZ: Methodology, Writing – original draft. TZ: Methodology, Writing – original draft. RX: Methodology, Writing – original draft. JZh: Methodology, Writing – original draft. TS: Methodology, Writing – original draft. SuZ: Methodology, Writing – original draft. CC: Methodology, Writing – original draft. SH: Methodology, Writing – original draft. XW: Methodology, Writing – original draft. YN: Methodology, Writing – original draft. KA: Data curation, Writing – original draft. QX: Supervision, Writing – review & editing. XZ: Funding acquisition, Writing – review & editing. GC: Conceptualization, Supervision, Writing – review & editing. WZ: Conceptualization, Writing – review & editing.

## Footnotes

^1^http://www.chictr.org.cn
